# Perceived facial happiness during conversation correlates with insular and hypothalamus activity for humans, not robots

**DOI:** 10.3389/fpsyg.2022.871676

**Published:** 2022-10-03

**Authors:** Thierry Chaminade, Nicolas Spatola

**Affiliations:** ^1^Institut de Neurosciences de la Timone, UMR 7289, Aix-Marseille Université-CNRS, Marseille, France; ^2^Center for Human Technologies, Istituto Italiano di Tecnologia, Genoa, Italy

**Keywords:** humanoid robot, social cognitive neuroscience, happiness, conversation, fMRI, insula, hypothalamus, amygdala

## Abstract

Emotional contagion, in particular of happiness, is essential to creating social bonds. The somatic marker hypothesis posits that embodied physiological changes associated with emotions and relayed to the brain by the autonomous nervous system influence behavior. Perceiving others’ positive emotions should thus be associated with activity in brain regions relaying information from and to the autonomic nervous system. Here, we address this question using a unique corpus of brain activity recorded during unconstrained conversations between participants and a human or a humanoid robot. fMRI recordings are used to test whether activity in key brain regions of the autonomic system, the amygdala, hypothalamus, and insula, is differentially affected by the level of happiness expressed by the human and robot agents. Results indicate that for the hypothalamus and the insula, in particular the anterior agranular region strongly involved in processing social emotions, activity in the right hemisphere increases with the level of happiness expressed by the human but not the robot. Perceiving positive emotions in social interactions induces local brain responses predicted by the contagion of somatic markers of emotions only when the interacting agent is a fellow human.

## Introduction

Human beings are intrinsically social creatures whose lives are intertwined with others. Engaged in any social interaction, we cannot help but gather information about others’ mental states, in particular their emotions that signal the quality of the ongoing interaction. Facial expressions convey many signals about people’s internal states, which are extremely relevant for smooth social interactions. Our capacity to take into account these signals allows us to adapt our behavior when interacting with others. One adaptation is emotional contagion, which describes the phenomenon that many components, including the autonomous nervous system’s response, of others’ emotions, are shared by the observer (Hatfield et al., 1993). Such contagion can even take place in the absence of explicit perception, as it has been found for pupil size (Harrison et al., 2006) and suggested for subtle facial blushing (Crozier, 2001).

Understanding brain mechanisms involved in emotional contagion during naturalistic social interactions is required for the advancement of second-person neuroscience (Redcay and Schilbach, 2019), which advocates the study of real time and reciprocal exchanges between individuals. The somatic marker hypothesis postulates that autonomic responses associated with specific emotions are re-enacted in response to certain stimuli (Damasio et al., 1996). As part of the mirroring involved in emotional contagion (Hatfield et al., 1993), such stimuli could be perceiving emotions from others, which would automatically entail re-enacting them, not only in the motor (Goldman, 2006) but also in the autonomic nervous system level. A controlled fMRI experiment showed that changes reduced the response in the anterior insula bilaterally as well as in the amygdala (Harrison et al., 2009).

The insular cortex and the amygdala (Damasio et al., 2000; Pessoa, 2017) are core brain areas involved in associating peripheral and central nervous systems. The hypothalamus plays an important role in endocrine signaling. These regions share many characteristics, such as being involved in core body functions such as homeostasis, ubiquitous in the vertebrate kingdom, and smaller in humans among primates relative to other subcortical (e.g., hippocampus) or cortical (e.g., prefrontal cortex) structures. It could be that, in most vertebrates, emotions have a direct relation to survival and thus play an important role in natural selection. For example, fear is associated with specific homeostatic and behavioral “fight-or-flight” responses essential for animal survival. In contrast, higher cognitive processes taking place in the cortex, in particular the prefrontal cortex, prevail in humans.

Investigating the mechanisms underlying the processing of others’ emotions during natural interactions is difficult given the incompatibility between the necessary experimental control in experimental research and the freedom associated with natural social behavior. Exceptional circumstances, such as intracerebral sampling of hormones in narcoleptic patients, clearly demonstrate that the amygdala physiology is influenced by social emotions (Blouin et al., 2013). Artificial agents provide an experimental tool to bypass this difficulty. Human-like robots that can produce facial emotions have been developed, either in mechanical form (Chaminade et al., 2010) or through a projection (Al Moubayed et al., 2012), allowing the development of new paradigms to better understand mechanisms involved in human social interaction (Chaminade, 2017). They are unique tools to dissociate perceptual, bottom-up processes that are automatic and irresistible (such as speech understanding) from top-down contextual processes that are influenced by a large number of factors, amongst which the perceiver’s mental states play a major role.

For instance, two fMRI studies comparing brain response to the passive viewing of humans’ and robots’ facial expressions of emotions reported reduced activity for the robot expressions of emotions in the left insula and right amygdala (Hortensius et al., 2018). Another fMRI study (Chaminade et al., 2010) found reduced activity in the hypothalamus when participants believed they were playing with a robot compared to a human, without even seeing their opponent (Chaminade et al., 2015). However, these studies are not sufficient to answer the crucial question: are these effects due to the nature of the agent, a dichotomic factor, or rather quantitative differences in the intensity of the social emotions conveyed by the interaction? Here, we investigate this question, important both for understanding natural human social cognition and for the future of human-robot interactions, by analyzing a unique fMRI corpus of human–human and human–robot face-to-face conversations (Rauchbauer et al., 2019).

Considering “the six canonical emotions” as distinct categories, following a long trend of research initiated by Charles Darwin (Darwin, 1872) and continued one century later by Paul Ekman (Ekman, 1974), we proposed that the main emotion for social bonding is the positive emotion “happiness.” In fact, positive emotions are reciprocally associated with social bonding, a robust relationship in most of our social interactions (see, for example, the strong linear correlation between reported happiness and the proportion of time spent with other people, Figure 1 in Quoidbach et al., 2019). Also, it has been proposed that “autism spectrum disorder (ASD) can be construed as an extreme case of social motivation” (Chevallier et al., 2012), meaning that abnormal positive appraisal of social interactions, a process referred to as “social motivation” that combines, at the behavioral level, the orienting toward social stimuli, the liking of social interactions, and the maintenance of social bonds, is responsible for a major disorder of social cognition.

At the brain level, our initial postulate is that the processing of facial expressions of emotions comprises at least two distinct mechanisms (Adolphs, 2002), as do many perceptual mechanisms involved in social interactions, such as the perception of faces or voices (Belin, 2017). First, a fast visual mechanism identifies the geometric configuration of facial features, usually described as norm-based coding of facial patterns elicited by muscular activations. These activations, also known as Facial Action Units, can indeed be used to characterize the emotions expressed (Ekman and Friesen, 1978). A later mechanism involves the representations of emotions embodied in the autonomous nervous system. This mechanism does not compute a norm-based coding of the sensory percept but, according to the somatic markers hypothesis (Damasio et al., 1996), it induces a response of the autonomic nervous system that spreads through the whole body. Brain structures at the interface of the central nervous system and the peripheral autonomous nervous system, including neuroendocrine signaling, should play a key role in this response.

In practice, we tested this hypothesis by automatically extracting facial happiness expressed by the human and robot agents and using it as a covariate in multivariate analyses of the signal in functional subregions of the insula, in two divisions of the amygdala, and in the hypothalamus, bilaterally. We hypothesized that should emotional resonance yield an autonomic reaction to positive emotions perceived by the participants during a natural conversation, the response in these particular brain structures would correlate with the level of happiness expressed by the human (the natural agent) but not the robot (the artificial agent).

## Materials and methods

As the analysis presented here is based on an existing available corpus described *in extenso* in previous publications (Rauchbauer et al., 2019, 2020), we will only summarize the information necessary to support the results and discussion, but readers are referred to the technical publications for more details.

### Data acquisition

**FIGURE 1:**
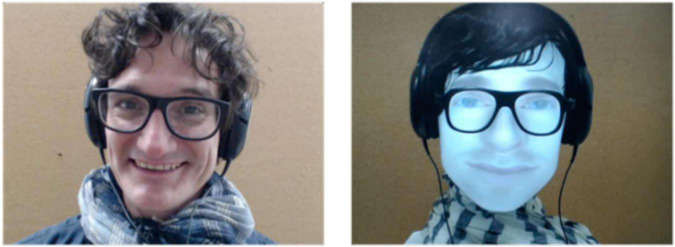
Snapshots from the live video feeds projected to the scanned participants, with the human (left) and robot (right) interlocutor.

When participants (*n* = 24, 17 women, μ = 26.76 years, σ = 7.96) arrived at the MRI center, one experimenter presented them with the cover story. To allow participants to have natural conversations despite the experimental setting, they were kept in the dark about the actual purpose of the experiment through a cover story. They were told that they were participating in a neuromarketing experiment. Specifically, a company asked neuroscientists to determine whether two people could guess the meaning of an upcoming advertising campaign simply by discussing the images used to illustrate it. The experimenter then introduced them to the agents with whom they would be discussing. The human partner was a confederate of the experimenter gender-matched with each participant, and the artificial agent was a back-projected conversational robotic head (Al Moubayed et al., 2012) with an appearance, voice, and accessories reminiscent of the natural agent (Figure 1). Only one version of the artificial agent was used per participant.

During functional magnetic resonance imaging (fMRI) acquisition, participants discussed online with these two agents alternatively while lying supine in the scanner and having their brain activity recorded. A BOLD signal (blood oxygen level dependent) was recorded in four sessions of fMRI acquisition, each lasting approximately 8 min. Each session comprised 6 experimental trials that proceeded as follows: a picture appeared for 8.3 s, then after a 3-s pause with a white fixation cross on a black background, a 1-min live conversation took place with either the human or the robot agent, alternatively. The participant and interlocutor could hear each other in real time, and the participant additionally saw a live feed of their interlocutor. All audios and videos available were recorded for further analysis. Other variables were recorded, such as the participant’s eye movements or heart rate, but these will not be discussed further here since they are not used in the current analysis. In total, each participant took part in twelve 1-min conversations with the human and twelve 1-min conversations with the robot.

Unbeknown to the participants, who believed the robot was autonomous, it was actually controlled remotely by the confederate acting as the human interlocutor. A set of pre-recorded answers stored as text snippets chosen based on a preparatory version of the experimental procedure were selected in real time by pressing virtual buttons on a touch-sensitive tablet. Responses were played using the voice synthesizer provided by the robot. Some answers were generic (e.g., “yes,” “no,” “maybe,” and “I do not know”), and others were specific to an image (“It is a yellow pear”) or to one of the two advertising campaigns (“Maybe it is a campaign to promote local fruits cultivation”). Importantly, emotional expressions by the robot were not actively controlled but happened as part of random movements encoded in the robot to increase its naturalness, except on very rare occasions when happiness expressions were used as non-verbal feedback.

### Data preparation

The processing of fMRI data followed standard procedures. The volumes acquired represent the BOLD signal in 2.5 × 2.5 × 2.5 cm^3^ voxels of the brain. Each volume contains 54 slices of 84 × 84 in-plane voxel and is recorded in 1.205 s. Pre-processing entails a correction of the temporal synchronization of the acquired slices, a realignment of the volumes of each session on the first one, and a correction of the deformations due to the local distortions of the magnetic field and participants’ movements. Normalization uses the DARTEL procedure (Ashburner, 2007) to put the imaged brains of all participants in the standard MNI space. The artifact detection tools (ART) were tested for any movement-related artifacts,^1^ and none were detected using the standard threshold of 2 mm. Several nuisance covariates were computed to eliminate motion artifacts, potential blood pulse, and respiration artifacts, which were highly relevant in a paradigm involving speech, as well as global gray matter signal, white matter activity, and cerebrospinal fluid activity to control global signal fluctuations unrelated to the task (TAPAS toolkit; Kasper et al., 2017).

**FIGURE 2:**
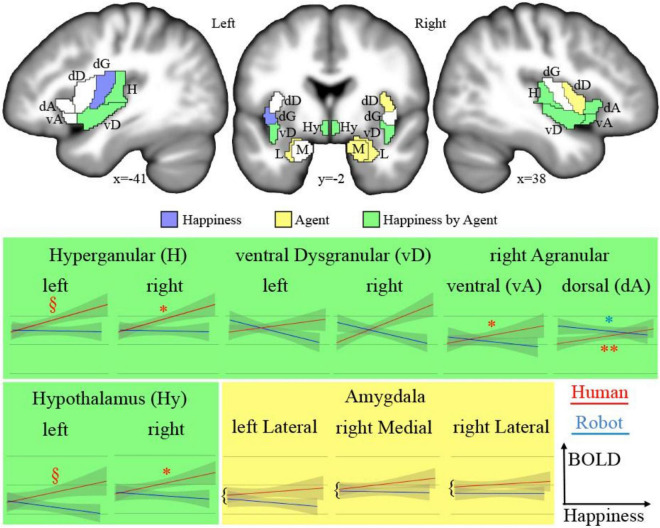
Top: Regions of interests (acronyms described in Table 1) overlaid on sections of the average of participants’ brains after normalization to MNI space. In blue are the areas showing a significant main effect of happiness; in yellow, a significant main effect of the type of agent; and in green, a significant interaction. Bottom: Plots of significant effects on BOLD response [arbitrary units (AU) after normalization] against the level of happiness expression for human (red) and robot (blue). The significant correlations are indicated as follows: ^**^p < 0.001, *p < 0.050, ^§^*p* < 0.100.

The analysis of fMRI data was first based on the general linear model implemented in SPM (Friston et al., 2007). Each trial was modeled as a single regressor, and the presentation of images before each discussion was modeled as a single regressor. We used a brain parcellation formed from functional and connectivity brain data, the Brainnetome atlas (Fan et al., 2016), so that the 246 regions of interest of the atlas represent sets of voxels that are homogeneous in terms of function. For each of the 24 trials (12 for the human and 12 for the robot) of each of the 24 participants, we extracted the average response across the 1-min duration using the MarsBAR toolbox (Brett et al., 2002). We then focused on regions of interest (ROIs) that belong to the insular and subcortical insular regions that form the core of the somatic emotional system. The insula can be parcellated according to a gradient of increasing granularity from front to back. Within the Brainnetome atlas numbering system, insular regions are (in left/right hemispheres) 165/166 [ventral agranular, (vA)] and 167/168 [dorsal agranular, (dA)] anteriorly, 169/170 [ventral dysgranular, (vD)] and 173/174 [dorsal dysgranular, (dD)] in intermediate location, and 171/172 [dorsal granular insula, (dG)] and 163/164 [hypergranular insula (H)] posteriorly. The amygdala is composed of regions 211/212 [medial part of the amygdala (M), roughly corresponding to the medial and basal nuclei] and 213/214 [lateral part of the amygdala (L), roughly corresponding to central and lateral nuclei; Saygin et al., 2011]. The activity of the hypothalamus [Hy] was also extracted using a mask previously developed (Wolfe et al., 2015), given that this key region for homeostasis is not featured in the Brainnetome atlas. Figure 2 shows all regions.

**TABLE 1:** Results of the statistical analysis.

MNI coordinates	Effect	Statistics			95% CI		
x, y, z		*T*	*p*	β	(Lower–Upper)		
**Insula**							
**Ventral agranular (vA)**							
Left –32, 14, –13	Agent	0.25	0.805	0.022	–0.207	–	0.266
	Happiness	–0.89	0.374	–0.040	–0.016	–	0.006
	Happiness*Agent	0.97	0.330	0.085	–0.005	–	0.016
Right 33, 14, –13	Agent	–1.03	0.302	–0.090	–0.346	–	0.107
	Happiness	0.54	0.587	0.024	–0.007	–	0.013
	Happiness*Agent	**2.12**	**0.035**	**0.185**	**0.001**	–	**0.021**
**Ventral agranular (dA)**							
Left –34, 18, 1	Agent	–1.50	0.134	–0.131	–0.454	–	0.061
	Happiness	1.56	0.121	0.070	–0.002	–	0.021
	Happiness*Agent	0.19	0.846	0.017	–0.011	–	0.013
Right 36, 18, 1	Agent	**–3.24**	**0.001**	**–0.281**	**–0.696**	–	**–0.170**
	Happiness	0.53	0.599	0.024	–0.009	–	0.015
	Happiness*Agent	**2.21**	**0.028**	**0.192**	**0.001**	–	**0.025**
**Ventral Dysgranular (vD)**							
Left –38, –4, –9	Agent	**–2.01**	**0.045**	**–0.175**	**–0.501**	–	**–0.006**
	Happiness	–0.92	0.356	–0.041	–0.017	–	0.006
	Happiness*Agent	**2.94**	**0.003**	**0.255**	**0.006**	–	**0.028**
Right 39, –2, –9	Agent	**–3.39**	**0.001**	**–0.290**	**–0.709**	–	**–0.189**
	Happiness	1.35	0.179	0.059	–0.004	–	0.020
	Happiness*Agent	**4.93**	**0.000**	**0.422**	**0.018**	–	**0.042**
**Dorsal Dysgranular (dD)**							
Left –38, 5, 5	Agent	–1.49	0.138	–0.130	–0.436	–	0.061
	Happiness	–0.22	0.830	–0.010	–0.013	–	0.010
	Happiness*Agent	0.82	0.414	0.072	–0.007	–	0.016
Right 38, 5, 5	Agent	**–2.70**	**0.007**	**–0.236**	**–0.566**	–	**–0.089**
	Happiness	0.32	0.752	0.014	–0.009	–	0.013
	Happiness*Agent	1.83	0.068	0.160	–0.001	–	0.021
**Dorsal Granular (dG)**							
Left –38, –8, 8	Agent	–1.09	0.277	–0.095	–0.373	–	0.107
	Happiness	**2.27**	**0.024**	**0.102**	**0.002**	–	**0.024**
	Happiness*Agent	1.34	0.180	0.117	–0.003	–	0.018
Right 39, –7, 8	Agent	0.04	0.966	0.004	–0.252	–	0.263
	Happiness	1.23	0.221	0.056	–0.004	–	0.019
	Happiness*Agent	–0.21	0.835	–0.018	–0.013	–	0.010
**Hypergranular (H)**							
Left –36, –20, 10	Agent	–0.36	0.716	–0.031	–0.307	–	0.211
	Happiness	**2.11**	**0.035**	**0.094**	**0.001**	–	**0.025**
	Happiness*Agent	**2.43**	**0.015**	**0.210**	**0.003**	–	**0.026**
Right 37, –18, 8	Agent	–1.07	0.287	–0.091	–0.408	–	0.121
	Happiness	**2.52**	**0.012**	**0.112**	**0.003**	–	**0.028**
	Happiness*Agent	**3.20**	**0.001**	**0.275**	**0.008**	–	**0.032**
**AMYGDALA**							
**Medial (M)**							
Left –19, –2, –20	Agent	1.40	0.162	0.063	–0.032	–	0.193
	Happiness	1.75	0.081	0.079	–0.001	–	0.019
	Happiness*Agent	0.55	0.582	0.048	–0.007	–	0.013
Right 19, –2, –19	Agent	**2.51**	**0.012**	**0.113**	**0.031**	–	**0.253**
	Happiness	0.81	0.418	0.036	–0.006	–	0.014
	Happiness*Agent	1.17	0.241	0.102	–0.004	–	0.016
**Lateral (L)**							
Left –27, –4, –20	Agent	**3.12**	**0.002**	**0.140**	**0.067**	–	**0.295**
	Happiness	–0.15	0.882	–0.007	–0.011	–	0.009
	Happiness*Agent	1.25	0.214	0.108	–0.004	–	0.016
Right 28, –3, –20	Agent	**3.34**	**0.001**	**0.149**	**0.079**	–	**0.306**
	Happiness	0.48	0.635	0.021	–0.008	–	0.012
	Happiness*Agent	0.54	0.587	0.047	–0.007	–	0.013
**Hypothalamus (Hy)**							
Left –3, –7, –8	Agent	–0.29	0.773	–0.025	–0.222	–	0.165
	Happiness	0.57	0.568	0.025	–0.006	–	0.011
	Happiness*Agent	**2.45**	**0.015**	**0.212**	**0.002**	–	**0.020**
Right 3, 7, 8	Agent	–0.24	0.815	–0.020	–0.220	–	0.173
	Happiness	1.15	0.250	0.051	–0.004	–	0.014
	Happiness*Agent	**2.34**	**0.020**	**0.202**	**0.002**	–	**0.020**

Significant effects (*p* < 0.05) are indicated in bold.

### Data analysis

Facial emotions were extracted automatically from the videos of the human and robot interlocutors using a freely available machine learning algorithm (Arriaga et al., 2019) available at https://github.com/oarriaga/face_classification. The algorithm uses a convolutional neural network to classify facial expressions into 7 classes (anger, disgust, fear, happiness, sadness, surprise, and neutral). According to its developers, it can achieve an accuracy of 66% in the Facial Emotion Recognition (FER-2013) dataset, which consists of manually labeled 35,887 48 × 48 pixels grayscale images. More precisely, the algorithm achieved a correct recognition of 87% of happy faces, with mislabeling below 5%, while the correct recognition was in the range of 41% (for fear) to 77% (for surprise) for the other emotions according to the confusion matrix. We verified that emotions depicted by the robotic device were correctly recognized by the algorithm and found correct detection for the happy and neutral emotions, which were the only facial expressions used in the recording of the corpus. In addition, the recordings used for this classification, 640 × 480 images of the full-screen face of the human or robot interlocutor looking directly at the camera, were particularly well-suited for this automatic classification, in contrast to the more variable faces, in terms of age, orientation, brightness, and occlusion, used for the development of the algorithm. Altogether, these arguments support the use of automatically recognized happy emotions in our analysis. In practice, the probability predicted for happy emotion was extracted frame by frame, then summed over all frames of a trial, therefore providing one happiness score for each trial.

Statistical analyses were performed in R using the package lme4. Multivariate models were used as the effect of each predictor (level of facial happiness, type of agent) was evaluated while holding constant the effect of the other predictors on the dependent variable. We introduced the happiness score obtained for each trial as a predictive continuous variable for all regions evaluated, with the nature of the agent as the categorical factor of interest. The identity of the participant and sessions were used as random variables. We used Holm’s correction for multiple comparisons (Holm, 1979) to control the family-wise error rate. Holm’s correction has been used in neuroimaging analyses when multiple comparison corrections for family-wise errors are too stringent given a large number of observations, in the analysis of functional MRI brain activation (Leong et al., 2018), but also in whole-body anatomy (Breznik et al., 2020). The models were selected in a backward process based on the restricted maximum likelihood.

## Results

As expected, there is a significant difference between the happiness extracted for the human and robot interlocutors (*t* = 21.08, *p* < 0.001, CI 95% [0.89, 2.76]), with higher scores for the human. This significant difference argues in favor of the multivariate linear model approach that allows us to evaluate independently each term’s unique contribution to the variation of the average BOLD signal per trial, therefore controlling for the significant difference in happiness between agents.

We tested the effect of the dichotomous factor, describing the nature, human or robotic, of the participant’s interlocutor (agent) and of the continuous happiness score (happiness), as well as the interaction between these two terms (happiness by agent) on the BOLD signal in the insula, amygdala, and hypothalamus ROIs. Statistical results for all ROIs are presented in Table 1 and illustrated in Figure 2 (top). The interaction term is particularly important as it indicates that the happiness score affects local brain response differently depending on whether the interlocutor is a human or a robot. This term reveals different but consistent patterns in the amygdala ROIs on the one hand, for which it is never significant, and in the insula and hypothalamus ROIs on the other hand, for which it is significant in many regions.

Results provided in Table 2 and illustrated in Figure 2 (bottom) indicate a significant positive correlation between BOLD signal and happiness for the human agent in two right hemisphere insula ROIs (dorsal agranular and hypergranular) as well as a trend for the left hypergranular region. In parallel, the analyses revealed a negative correlation for the robot agent in the right ventral and dorsal agranular insula ROIs. The correlation was positive for the human agent in the hypothalamus (significant on the right; trend on the left) and not significant for the robot.

**TABLE 2:** Effect of happiness on BOLD signal calculated separately for the human and robot agent in ROIs having a significant agent * happiness interaction.

Regions	Human			95% CI			Robot			95% CI		
	*t*	*p*	β	(Lower	–	Upper)	*t*	*p*	β	(Lower	–	Upper)
Insula												
Ventral agranular (vA), right	1.46	0.147	0.092	–0.004	–	0.027	**–2.68**	**0.008**	**–0.167**	**–0.038**	–	**–0.006**
Dorsal agranular (dA), right	**4.34**	**<0.001**	**0.265**	**0.021**	–	**0.055**	**–2.59**	**0.010**	**–0.160**	**–0.038**	–	**–0.005**
Ventral dysgranular (vD), left	1.55	0.121	0.098	–0.003	–	0.027	–1.05	0.295	–0.066	–0.024	–	0.007
Ventral dysgranular (vD), right	0.43	0.667	0.027	–0.012	–	0.019	–0.72	0.470	–0.046	–0.022	–	0.010
Hypergranular (H), left	1.90	0.059	0.119	–0.001	–	0.028	–1.10	0.271	–0.070	–0.023	–	0.006
Hypergranular (H), right	**2.02**	**0.044**	**0.127**	**0.000**	–	**0.033**	–1.15	0.252	–0.072	–0.028	–	0.007
Hypothalamus (Hy), left	1.93	0.054	0.121	0.000	–	0.027	–1.50	0.136	–0.094	–0.020	–	0.003
Hypothalamus (Hy), right	**2.41**	**0.017**	**0.150**	**0.003**	–	**0.029**	–0.86	0.391	–0.054	–0.018	–	0.007

Significant effects (*p* < 0.05) are indicated in bold.

## Discussion

Using a multimodal corpus of human–human and human–robot conversations in which participants’ brain activity was recorded with fMRI, we investigated how the emotion expressed by the interlocutor influences activity in central brain areas interacting with the peripheral autonomous nervous system, looking for evidence of emotional contagion at the somatic level. More precisely, the facial expression of happiness of the interlocutor, human or robot, was quantified automatically for each trial and used as a continuous variable against the BOLD response in subregions of the hypothalamus, the amygdala, and the insula. The rest of the discussion focuses on brain regions in which the interaction term between the happiness score and the nature of the agent is significant, as it identifies areas in which the response to the emotion depends on the nature of the agent expressing this emotion. This interaction term was significant in the hypothalamus bilaterally as well as in subregions of the insula but not in the amygdala. Particularly interesting was the activity in the right anterior insula, which increased with happiness expressed by the human, but not the robot, face.

The expression of happiness was significantly higher for humans than for robots. This was expected given that the artificial device used in this experiment (refer to Figure 1, left) had clear limitations in the expression of emotion, not only visually (smiles had to be controlled explicitly, which was extremely rare) but also auditorily (voice intonations could not be controlled). In contrast, humans behaved naturally, regularly engaging in jokes and laughter. Therefore, humans expressed quantitatively higher levels of happiness than robots. Multivariate models were used to control for the potential statistical bias that could result from this intrinsic difference in the analysis of the BOLD response in the insula, the amygdala, and the hypothalamus, with the nature of the agent and level of happiness as factors of interest.

In the amygdala, only the main effect of the agent was statistically significant, with increased BOLD response during human trials in 3 out of the 4 subregions (lateral amygdala bilaterally; medial amygdala in the left hemisphere). As explained in the “Introduction” section, emotions are a complex construct and difficult to investigate, in particular during natural interactions. Fear has been the focus of most attention in animal models thanks to the paradigm of fear conditioning that allows investigating this emotion without verbal assessment of the participant’s feelings. Thanks to these animal studies, it is well known that the amygdala is necessary for fear conditioning, which takes the form of long-term potentiation within fear circuits. Interestingly, the lateral parts of the amygdala depict a larger effect of agent (*t*-scores > 3 in the two hemispheres) than the medial division (*t*-scores < 3)—the latter is associated with visceral inputs compared to the former being associated with sensory, including visual and auditory, inputs (LeDoux, 2007) that carry the emotional information in the present experiment. The main effect of the agent in the amygdala, corresponding to an increased response for the human compared to the robot agent, could reflect the large differences in the facial expressions and voice intonations of the two agents. In fact, by directly measuring the levels of two hypothalamic neuropeptides in the amygdala, Blouin et al. (2013) reported an increase in hypocretin release in the amygdala associated with positive emotions and social interactions.

In many regions of the insula (colored green in Figure 2, top) and in the hypothalamus bilaterally, the facial happiness expressed by the interlocutor affected the brain activity differently depending on the nature of the agent displaying the emotion. In regions with significant interactions, plotting the BOLD response as a function of happiness (depicted with green background in Figure 2, bottom) always resulted in similar profiles, with a positive correlation of the response for the human agent and no effect or a decrease for the human agent. More precisely, increases for the human agent were statistically significant in the right hemisphere for the hypothalamus, the hypergranular, and the dorsal agranular insula regions, and the decrease for the robot was significant for the right dorsal agranular insula region only. The insula and hypothalamus are closely related to embodied emotional processing and are known to be influenced by social context (Bartz et al., 2011; Bernhardt and Singer, 2012). The responses of these regions could be interpreted as central somatic markers of emotions, the former receiving visceral-somatic signals (Uddin et al., 2017) and the latter secreting, in the brain and in the blood circulation, neuropeptides including oxytocin associated with social bonding (Chevallier et al., 2012) and hypocretin discussed previously (Blouin et al., 2013). In particular, the anterior part of the insula (Morel et al., 2013), which includes the right dorsal agranular region where we report a positive correlation of brain activity with happiness expression by humans and a negative correlation for robots’ expression, contains Von Economo Neurons that have a fundamental role in subjective feelings (Craig, 2009). We speculate that this inversion of correlation not only signals the expected empathy for fellow humans, as an increase in the happiness displayed by the interlocutor triggers an increase in the local response, but also the repulsion for imperfect human-like robots postulated by the Uncanny Valley hypothesis (Mori et al., 2012).

The results of the correlation analysis provide important insights into how the regions under scrutiny are involved in social cognition. First, they support the dominance of the right hemisphere in emotional processing, which was proposed to be related to the asymmetry of the autonomous nervous system involved in homeostasis (Craig, 2005), as no significant correlation was found in the left hemisphere. In the hypothalamus, it is interesting that the brain response is similar for the two agents at the lowest levels of happiness expressed, and the difference increases with the expression of emotion, a strong argument in favor of the role of this region in the building of empathetic bonds exclusively with conspecifics, possibly through the release of oxytocin. In the insula, the results provide an interesting parcellation with regard to social cognition. The anterior agranular regions, known to be involved in socioemotional processes, in fact, depict, in the right hemisphere, the responses expected for this function. Dorsal regions, associated with cognitive functions, do not show a clear pattern in relation to the experimental conditions. Finally, results suggest an involvement of posterior hypergranular and ventral dysgranular regions, associated with sensorimotor and chemical senses, respectively (Uddin et al., 2017), in social interactions, in line with the view of embodied cognition. Differences in the patterns of these two regions are of potential interest, but the absence of strong statistical significance precludes interpretations relying exclusively on social cognition explanations. For example, increased happiness by the human interlocutor could cause increased verbalization, leading to a stronger reliance on sensorimotor processes.

## Limitations

An important limitation pertains to the automatic extraction of the happiness scores from the videos. The convolutional neural networks were trained and tested with human faces (Arriaga et al., 2019) but not validated with the robotic device we used. We ran the algorithm on *ad hoc* video files in which the different facial emotions had been controlled by the experimenter and found that neutral and happy emotions (i.e., smiles) were correctly recognized, while there were attribution errors on the other, in particular negative, emotions. Importantly, as noted previously, the algorithm makes the same type of errors on human images. Finally, it is important to keep in mind that the robot’s facial emotions, as well as its utterances and intonations, are fully controlled and therefore show less variation from trial to trial. Given all these reasons, we are confident that the rating of happiness on the robot’s face is objective as smiles are the only facial expressions used when recording the corpus. Finally, it should be added that, from a social cognition standpoint, subjective annotation or rating of a robot’s facial expression of emotion is associated with other issues that are research questions of their own; for example, how does the knowledge and vision that the emotion is displayed by an artificial agent cause a top-down bias in the way the emotion is being perceived by a human observer?

The robotic device used in this experiment had clear limitations in the expression of emotion, both visually and orally. Even though there was no expression of negative emotions, its expression of happiness was restricted to a limited number of smiles, and we cannot rule out that the limited positive emotions depicted by the robot precluded creating more social bonds with the humanoid interlocutor. Efforts are underway to endow the robot with more natural facial expressions of emotion to complement the current corpus and address this question more directly. The statistical analysis was devised to take into account this unbalances between the human and robot agents. Three comments on the results comfort the experimental paradigm and the statistical analysis used here.

First, there are statistically significant results, demonstrating that despite difficulties related to the experimental approach chosen, that is, recording brain activity during unconstrained behaviors and *post hoc* relating the activity to conversational characteristics extracted from the recorded behaviors, the analysis allows to reveal brain correlates associated with these characteristics of natural interactions. This is particularly important as such approaches could become a hallmark of second-person neuroscience if its goal is to investigate natural interactions. Second, in all of the regions investigated (sometimes only in one hemisphere), activity is significantly affected by the characteristic under investigation, here the happiness expressed by the human and robot interlocutor. This also supports our choice of regions to investigate the perception of emotions in relation to emotional contagion and homeostasis. Finally, the finding that not only the factor describing the nature of the interlocutor, human or robot, but also the interaction term involving the more subtle happiness score estimated automatically, yields statistically significant results is in direct agreement with one central assumption of this project, that during natural social interactions, the nature of the interacting agent, human or robot, is an outstanding factor influencing the behavior and underlying neural correlates.

## Conclusion

The analysis presented here demonstrates how robots can help understand how the human brain processes social information during natural interactions. Focusing on brain regions at the interface between the central and autonomous nervous system—insula, amygdala, and hypothalamus—the results demonstrate that emotional resonance, in relation to homeostasis and the somatic markers hypothesis, is differently affected by the facial expressions of happiness according to the nature of the interlocutor. Positive correlations with the level of happiness expressed by the human interlocutor were found in two key regions for social bonding, namely, the anterior insula and hypothalamus. These correlations are particularly important because they are not found for the robot agent, illustrating the importance of artificial agents to investigate the specificities of human social interactions.

The development of robots capable of complex social interactions requires an objective assessment of their social competence. Despite the limitations of the current analysis, its results illustrate how crucial interdisciplinarity—bringing together social cognitive neuroscience, automatic analysis of complex behaviors, and the design of interactive agents—is for social robotics. Considering the fundamental role of emotions in social interactions, the results directly question the acceptability of human-like robots as natural partners. Thus, in addition to providing insights into the brain correlates of human social interactions, such paradigms also help answer the question: “To what extent can human-like robots be treated as social agents?”

## Data availability statement

Publicly available datasets were analyzed in this study. This data can be found here: Transcribed linguistic data can be found on Ortolang (https://hdl.handle.net/11403/convers). fMRI raw data can be found on OpenNeuro (https://openneuro.org/datasets/ds001740).

## Ethics statement

The studies involving human participants were reviewed and approved by the Comité de Protection des Personnes Sud Méditerrannée 1 2016-A01008-43. The patients/participants provided their written informed consent to participate in this study. Written informed consent was obtained from the individual(s) for the publication of any potentially identifiable images or data included in this article.

## Author contributions

TC provided the corpus. TC and NS analyzed the data and wrote the manuscript. Both authors contributed to the article and approved the submitted version.

## Footnotes

1
www.nitrc.org/projects/artifact_detect
